# Inflammation-associated microRNA changes in circulating exosomes of heart failure patients

**DOI:** 10.1186/s13104-017-3090-y

**Published:** 2017-12-19

**Authors:** Faheemullah Beg, Ruizhong Wang, Zeb Saeed, Srikant Devaraj, Kamalesh Masoor, Harikrishna Nakshatri

**Affiliations:** 1 Department of Internal Medicine, IU School of Medicine, Indianapolis, IN USA; 2 Department of Surgery, IU School of Medicine, C218C, 980 West Walnut St., Indianapolis, IN 46202 USA; 3 Center for Business and Economic Research, Ball State University, Muncie, IN USA; 4 Department of Cardiology, Richard L Roudebush VAMC, Indianapolis, IN USA; 5 Department of Biochemistry and Molecular Biology, IU School of Medicine, C218C, 980 West Walnut St., Indianapolis, IN 46202 USA; 6 Richard L. Roudebush VA Medical Center, Indianapolis, IN USA

**Keywords:** Heart failure, MicroRNA, Exosomes, Biomarkers

## Abstract

**Objective:**

MiR-486 and miR-146a are cardiomyocyte-enriched microRNAs that control cell survival and self-regulation of inflammation. These microRNAs are released into circulation and are detected in plasma or in circulating exosomes. Little is known whether heart failure affects their release into circulation, which this study investigated.

**Results:**

Total and exosome-specific microRNAs in plasma of 40 heart failure patients and 20 controls were prepared using the miRVana Kit. We measured exosomal and total plasma microRNAs separately because exosomes serve as cargos that transfer biological materials and alter signaling in distant organs, whereas microRNAs in plasma indicate the level of tissue damage and are mostly derived from dead cells. qRT-PCR was used to quantify miR-486, miR-146a, and miR-16. Heart failure did not significantly affect plasma miR-486/miR-16 and miR-146a/miR-16 ratio, although miR-146a/miR-16 showed a trend of elevated expression (2.3 ± 0.79, p = 0.27). By contrast, circulating exosomal miR-146a/miR-16 ratio was higher in heart failure patients (2.46 ± 0.51, p = 0.05). miR-146a is induced in response to inflammation as a part of inflammation attenuation circuitry. Indeed, Tnfα and Gm-csf increased miR-146a but not miR-486 in the cardiomyocyte cell line H9C2. These results, if confirmed in a larger study, may help to develop circulating exosomal miR-146a as a biomarker of heart failure.

## Introduction

Heart failure with reduced ejection fraction (EF) is a major cardiovascular disease and contributes significantly to healthcare expenditure in the United States [1]. Currently, five million Americans are affected by heart failure and an additional 500,000 are diagnosed each year [2]. Five-year survival rate from heart failure is 50% [2]. Persistent aberrant interplay of various components of the immune system could lead to heart failure [3]. Many changes in the composition of biofluids after heart failure could result from damaged heart tissue or due to immune and non-immune-dependent systemic response to heart failures. These changes in biofluids can be exploited as biomarkers of disease course and response to therapy.

MicroRNAs (miR) are a class of short RNA molecules, which negatively regulate gene expression by targeting the 3′ untranslated region of specific mRNA [4]. MicroRNAs in circulation have been studied as biomarkers of cardiac pathophysiology [5, 6]. MicroRNAs from cells are released passively into circulation by damaged tissue or actively through exosomes [7]. MicroRNAs in circulation are predominantly exosomes/microvesicles-free and likely byproducts of dead cells without any biological activity [8]. By contrast, microRNAs in exosomes are transferred into cells in distant organs and actively modulate signaling in recipient cells [9, 10]. Because both free and exosomal microRNAs are relatively stable in circulation, circulating microRNAs are being developed as biomarkers of various diseases [11, 12]. We demonstrated that circulating microRNAs, particularly miR-486, serve as biomarkers of systemic effects of cancer [13].

This study was initiated to determine whether miR-486 serves as a biomarker of heart failure. Previous studies have shown elevated miR-486 in the plasma of patients with acute myocardial infarction [14] and coronary artery disease [15]. However, circulating level of miR-486 in patients with chronic heart disease is unknown. miR-486 is expressed predominantly in heart and muscle [16] and targets proteins in myogenesis (MyoD), myotube survival/differentiation (DOCK3/PTEN/AKT), cardiomyocyte survival (PI3K/PTEN/pAKT), and cardiac progenitor cell proliferation networks [16–19]. miR-486 is an integral part of myocardial homeostasis because it negatively regulates PIM1 kinase, which is essential for cycling of cardiac progenitor cells [16, 18, 19].

We included miR-146a in our study because heart failure is associated with inflammation, which induces miR-146a [20]. Induced miR-146a targets the same inflammatory molecules to attenuate inflammation. miR-146a has a cardiomyocyte-protective function as exposure to miR-146a increased cardiomyocyte viability and protection against oxidant stress [21, 22]. The purpose of this pilot study was to establish whether the levels of these two microRNAs are affected in heart failure.

## Main text

### Study population

We recruited 40 patients from outpatient cardiology clinic and inpatient medicine service at Richard L. Roudebush VA Medical Center, Indianapolis, between August-2015 and February-2016 (Table 1). Indiana University IRB approved the study. Written consent was obtained from all subjects. Inclusion criteria included an EF < 35% documented on most recent echocardiogram or NYHA class III or IV symptoms based on the assessment of patient’s primary cardiologist. Exclusion criteria included patients with a current or previous history of malignancy or inflammatory condition. We also recruited 20 veterans without a diagnosis of congestive heart failure or malignancy as controls. Demographics, clinical history, co-morbidities, echocardiographic and laboratory data were collected from electronic medical records. Almost all study participants were males with whites being the predominant ethnic group. Mean age and BMI were lesser in the control group. The incidence of hypertension, diabetes, coronary artery disease, and chronic kidney disease was significantly higher in the cases compared to controls (p < 0.05). The prevalence of alcohol and drug use was not significantly different between two groups. Patients with heart failure had a mean EF of 22.2 ± 7.2%. Ischemia was most likely underlying etiology of heart failure (47.5%). Some degree of diastolic dysfunction was reported on 25% of the echocardiograms (Table 2). The majority of the patients with heart failure were receiving guideline-directed medical therapy based on the etiology of heart failure and extent of depression in EF.

**Table 1:** Characteristics of study population at baseline

	Cases	Controls	Overall	p value
Number	40	20	60	
Age—year (SD)	66.5 (10.5)	60.6 (15.7)	64.5 (12.6)	0.09
Sex—no. (%)				0.16
Male	40/40 (100%)	19/20 (95%)	59/60 (98%)	
Race—no. (%)				
White	31/40 (78%)	16/20 (80%)	47/60 (80%)	0.82
African American	9/40 (22%)	3/20 (15%)	3/20 (15%)	0.50
Others		1/20 (5%)	1/20 (5%)	0.16
Body mass index (SD)	30.4 (7.4)	28.8 (6.1)	29.9 (7.0)	0.40
Medical history—no. (%)				
Hypertension	32/40 (80%)	11/20 (55%)	43/60 (72%)	0.04
Type 2 diabetes	23/40 (58%)	3/20 (15%)	26/60 (43%)	0.01
Coronary artery disease	23/40 (58%)	2/20 (10%)	25/60 (42%)	0.00
Chronic kidney disease	21/40 (53%)	1/20 (5%)	22/60 (37%)	0.00
Alcohol abuse	3/40 (8%)	1/20 (5%)	4/60 (7%)	0.72
Drug use	3/40 (8%)	2/20 (10%)	5/60 (8%)	0.74
Medications—no. (%)				
Aspirin	34/40 (85%)	6/20 (30%)	40/60 (67%)	0.00
Beta-blocker	38/40 (95%)	7/20 (35%)	45/60 (75%)	0.00
ACE/ARB	35/40 (88%)	2/20 (10%)	37/60 (62%)	0.00
Spirinolactone	16/40 (40%)	0/20 (0%)	16/60 (27%)	0.00
Statin	34/40 (85%)	9/20 (45%)	43/60 (72%)	0.00

**Table 2:** Characteristics of heart failure patients

Variables	
Ejection fraction	22.2 ± 7.2% (range 10–35%)
Presence of diastolic dysfunction	
None	30 (75%)
Stage 1 or 2	06 (15%)
Stage 3 or 4	04 (10%)
Etiology of heart failure	
Ischemic	19 (47.5%)
Non ischemic	14 (35.0%)
Unknown	07 (17.5%)

All values are expressed as N (percent of total cases)

Plus minus values are mean ± SD

### Sample processing

Total miRNAs from 200 µl of plasma samples were prepared using the miRVana miRNA isolation kit (AM1561, ThermoFisher Scientific). Total exosome isolation kit (ThermoFisher Scientific, Cat# 448445) was employed to extract exosomes from 200 μl plasma samples and miRNAs were isolated using the exosome RNA isolation kit (cat# 4478545). Exosome isolation kit has several quality control steps including precipitation of exosomes followed by wash and centrifugation steps to ensure purity of exosomes and quality of exosomes are similar to ultracentrifugation method [23]. Quantitative Reverse Transcription Polymerase Reaction (qRT-PCR) was used to quantify miR-486, miR-146a, and miR-16. Primers for qRT-PCR were purchased from ThermoFisher (hsa-miR-486-5p, Cat#001278; hsa-miR-146a-5p, Cat#000468; hsa-miR-16-5p, Cat#000391). The expression levels of miR-486 and miR-146a were normalized to miR-16 using the 2^−ΔΔCt^ method. Ratios between miR-486/miR-16 and miR-146a/miR-16 were calculated because there is no consensus on a microRNA that can be used as a normalization control. We had shown that the most commonly used U6 small RNA is not a reliable normalization control for circulating total microRNAs [24]. Similar concern has recently been raised regarding normalization controls for exosomal microRNAs [25]. We selected miR-16 for normalization because it is present abundantly in circulation and likely to be least variable as suggested by a previous study [26].

### Statistical analysis

The primary null hypothesis for this study was that there would be no difference in miR-146a and miR-486 levels between patients with heart failure and controls. The sample size was determined based on results of a previous study measuring circulating miR-486 levels in breast cancer patients. Using a two-sided Wilcoxon rank sum test, 14 subjects were needed in each group to ensure a power of 90%, with an alpha of 0.05. However, because of absence of data in patients with cardiac conditions, 40 subjects with disease and 20 healthy individuals were recruited. All continuous variables were expressed as mean ± SEM. Normalized expression levels of miR-486 and miR-146a were converted to fold-changes. Wilcoxon signed ranked test was used for comparison between groups. A p value of < 0.05 was considered to indicate statistical significance. SPSS 22.0 (Statistical Package for the Social Sciences, Chicago) and STATA 14.1 (StataCorp. 2015. Stata Statistical Software: Release 14. College Station, TX: StataCorp LP) were used for statistical analysis.

### Differences in microRNA levels between heart failure patients and controls

**Fig. 1:**
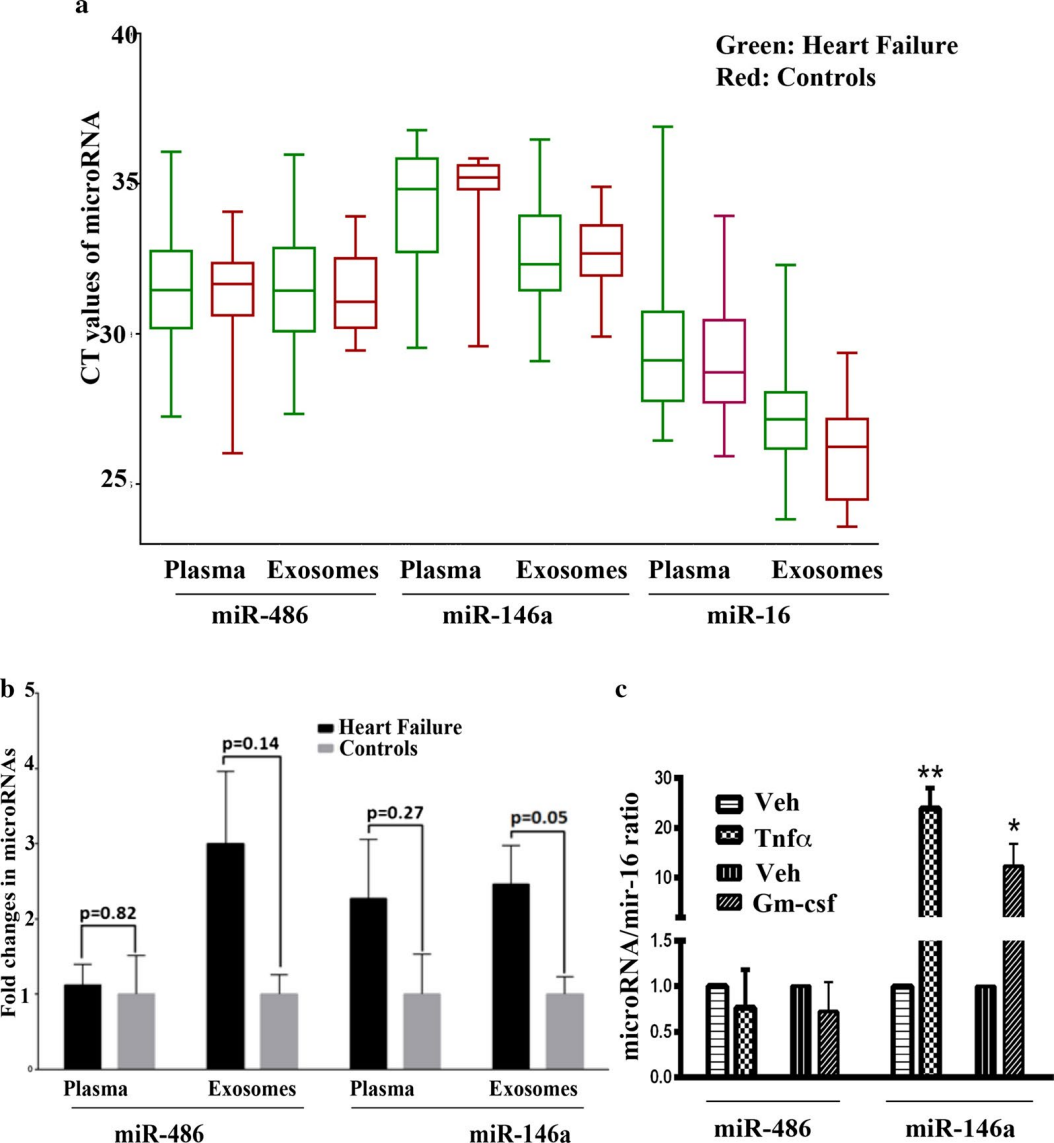
**a** Raw CT values of microRNA measurements in plasma and exosomes. **b** Levels of miR-486 and miR-146a in plasma and exosomes of patients experiencing heart failure (n = 40) compared to healthy controls (n = 20). Fold changes indicate ratio between indicated test microRNA versus abundantly and ubiquitously expressed/released miR-16. **c** The effects of Tnfα and Gm-csf on miR-146a and miR-486 expression in H9C2 cardiomyocytes. Cells maintained in DMEM plus 10% FBS were treated with Tnfα and Gm-csf at a concentration of 10 ng/ml for 24 h and microRNA levels were measured. miR-146a/miR-16 (n = 4, *p < 0.05, control vs. cytokine treatment) and miR-486/miR-16 ratio is presented

Raw CT values for each of the microRNAs are represented in Fig. 1a. Average fold-change for plasma levels of miR-486 and miR-146a showed a trend towards elevated expression in patients with heart failure (1.1 ± 0.27 and 2.3 ± 0.79 respectively) but was not statistically significant (0.82 and 0.14 respectively) (Fig. 1b). Circulating exosomes of heart failure patients contained a significantly elevated levels of miR-146a compared to controls (2.46 ± 0.51, p = 0.05). A similar trend was noted with exosomal miR-486 (3.0 ± 0.95, p = 0.14). Due to small number of cases, subgroup analysis based on the degree of EF suppression, medical comorbidities or types of therapy was not performed.

### Inflammatory cytokines induced miR-146a but not miR-486 in cardiomyocytes

To determine whether chronic inflammation associated with heart failure affects miR-146a and miR-486 expression in cardiomyocytes, we incubated the rat heart myoblast cell line H9C2 with Tnfα and Gm-csf (10 ng/ml) for 24 h and measured miR-146a/miR-16 and miR-486/miR-16 ratio. Both cytokines increased miR-146a but not miR-486 (Fig. 1c).

MicroRNAs have a role in almost all aspects of cardiac pathophysiology [5]. A number of studies have examined circulating levels of microRNAs, including cardiomyocyte-enriched microRNAs, in plasma or serum of patients with acute heart failure. However, not many microRNAs are consistently changed across studies in patients with heart failure with the exception of cardiac-specific miR-208. miR-208, which is involved in heart development, is upregulated in heart as well as in circulation upon acute myocardial infarction [27–31] and coronary artery disease [32]. A recent review emphasized the need for a larger clinical trial to develop clinically usable circulating microRNA biomarkers [6]. As the first step in this direction, we took a slightly different approach by measuring both free and exosome-encapsulated circulating microRNAs. Free circulating microRNA levels may show marked variability over time and likely dependent on the rate of tissue repair, which may be affected by both age and comorbidity [8]. By contrast, exosomal microRNAs reflects overall health status, need not have to originate from the damaged tissue, and could account for exosomes released by hematopoietic cells in circulation. Thus, this dual measurement permits detection of microRNAs released into circulation by dying cells and by other cell types in response to heart failure. Our study showed a significantly higher level of miR-146a in exosomes but not in plasma of patients with heart failure compared to controls. Previous studies have shown elevated plasma levels of miR-146a in patients with continuous flow left ventricular assist devices and in patients with peripartum cardiomyopathy (but not in patients with dilated cardiomyopathy) compared to healthy controls but there has been limited efforts in measuring exosomal levels of this microRNA in heart failure patients [33, 34]. To our knowledge, there is only one study that examined microRNAs in circulating exosomes in canine models of heart failure [35].

Chronic low-grade inflammation in patients with heart failure has been known [3, 36]. One crucial player in this chronic inflammation is the feed-forward loop involving Nuclear Factor-κB (NF-κB), which increases pro-inflammatory cytokines such as TNF-α, IL-1, IL-6, and IFN-γ and these cytokines, in turn, activate NF-κB [37]. Previous studies have shown NF-κB-inducible expression of miR-146a, which negatively effects IL-1 and TNF-α receptors to attenuate inflammation [38]. Consistent with this possibility, a recent study has shown cardioprotective effects of exosomal miR-146a derived from cardiac progenitor cells [39]. Thus, the increased exosomal level of miR-146a levels in heart failure patients is likely attributable to the interplay of various components of the inflammatory system in pathogenesis of heart failure.

Similar to miR-146a, miR-486 is a central component of the inflammatory hub as NF-κB reduces the levels of muscle-specific transcription factor MyoD, which regulates miR-486 expression in skeletal muscle [17, 40]. Apart from MyoD, myocardin-related transcription factor (MRTF) positively regulates miR-486 expression [16]. MRTF is a part of the inflammatory network and balance between the activity of MRTF and MyoD under inflammatory condition determines overall miR-486 expression levels [41]. Our patient population showed a trend towards the elevated expression of miR-486 levels, which is consistent with other studies showing elevated circulating miR-486 in patients with acute myocardial infarction and coronary aortic disorders [14, 15]. Sample size may be a reason our results did not reach statistical significance.

### Limitations

First, our sample size was small, although power calculations based on our previous study in breast cancer patients indicated sufficient power [13]. However, the aim of this study was to establish changes in levels of these heart and muscle-enriched microRNAs in patients with heart failure such that studies with much larger patient population can be planned in future. Second, our study didn’t compare microRNA levels with currently used biomarkers (BNP, NT-pro-BNP and Troponin). This was not included in the study initially because these markers are reflective of other pathways of heart failure namely myocyte stress and injury but not inflammation. Also, comparison studies involving hundreds if not thousands of patients would be required, if these markers are to be considered for clinical use. Third, these microRNA levels may be affected by other pathologies and their underlying mechanisms that we haven’t learned yet. Since the majority of patients with heart failure have other morbidities (diabetes, hypertension and/or renal failure) compared to healthy controls (p < 0.05), these confounding comorbidities, treatments, and as well as duration of the these comorbidities may affect circulating microRNA levels. Since these co-morbidities and heart failure co-exist in majority of patients, obtaining samples from patients suffering from only chronic heart failure may not be practical. Variations between patients with respect to time lapsed between initial diagnosis of heart failure and sample collection can additionally impact data interpretations Finally, microRNAs were measured at one time-point, which may fluctuate during the progression of the disease and during acute exacerbations. Thus, larger longitudinal studies involving measurement of microRNAs at multiple intervals starting from initial diagnosis, stable disease, and exacerbation may help to understand the fluctuation of levels with changes in disease state.

## Abbreviations

AKT: thymoma viral proto-oncogene 1
BMI: body mass index
Gm-csf: granulocyte macrophage colony stimulating factor
DMEM: Dulbecco’s Modified Essential Media
DOCK3: dedicator of cytokinesis 3
EF: ejection fraction
FBS: fetal bovine serum
IFNγ: interferon gamma
IL-1: interleukin one
IRB: institutional review board
miR: microRNA
MRTF: myocardin-related transcription factors
MyoD: myogenic differentiation 1
NYHA: New York Heart Association
PI3K: phosphoinositide-3-kinase
PIM1: proviral integration site 1
PTEN: phosphatase and tensin homolog
qRT-PCR: quantitative reverse transcription polymerase chain reaction
SEM: standard error of the mean
Tnfα: tumor necrosis factor alpha
